# Prebiotic mechanisms of resistant starches from dietary beans and pulses on gut microbiome and metabolic health in a humanized murine model of aging

**DOI:** 10.3389/fnut.2023.1106463

**Published:** 2023-02-07

**Authors:** Saurabh Kadyan, Gwoncheol Park, Prashant Singh, Bahram Arjmandi, Ravinder Nagpal

**Affiliations:** Department of Nutrition and Integrative Physiology, College of Health and Human Sciences, Florida State University, Tallahassee, FL, United States

**Keywords:** resistant starch, prebiotics, pulses, microbiota, gut dysbiosis

## Abstract

Dietary pulses, being a rich source of fiber and proteins, offer an ideal and inexpensive food choice for older adults to promote gut and metabolic health. However, the prebiotic effects of dietary pulses-derived resistant starches (RS), compared to RS from cereals and tubers, remain relatively underexplored, particularly in context to their gut modulatory potential in old age. We herein investigate the prebiotic effects of pulses-derived RS on the gut microbiome and intestinal health in aged (60-week old) mice colonized with human microbiota. C57B6/J mice were fed for 20 weeks with either a western-style high-fat diet (control; CTL) or CTL diet supplemented (5% w/w) with RS from pinto beans (PTB), black-eyed-peas (BEP), lentils (LEN), chickpeas (CKP), or inulin (INU; reference control). We find that the RS supplementation modulates gut microbiome in a sex-dependent manner. For instance, CKP enriched α-diversity only in females, while β-diversity deviated for both sexes. Further, different RS groups exhibited distinct microbiome differences at bacterial phyla and genera levels. Notably, LEN fostered Firmicutes and depleted Proteobacteria abundance, whereas Bacteroidota was promoted by CKP and INU. Genus *Dubosiella* increased dominantly in males for all groups except PTB, whilst *Faecalibaculum* decreased in females by CKP and INU groups. Linear discriminant analysis effect size (LEfSe) and correlational analyzes reveal RS-mediated upregulation of key bacterial genera associated with short-chain fatty acids (butyrate) production and suppression of specific pathobionts. Subsequent machine-learning analysis validate decreased abundance of notorious genera, namely, *Enterococcus*, *Odoribacter*, *Desulfovibrio*, *Alistipes* and *Erysipelatoclostridium* among RS groups. CKP and LEN groups partly protected males against post-prandial glycemia. Importantly, RS ameliorated high-fat diet-induced gut hyperpermeability and enhanced expression of tight-junction proteins (claudin-1 and claudin-4), which were more pronounced for LEN. In addition, IL10 upregulation was more prominent for LEN, while TNF-α was downregulated by LEN, CKP, and INU. Together, these findings demonstrate that RS supplementation beneficially modulates the gut microbiome with a reduction in gut leakiness and inflammation, indicating their prebiotic potential for functional food and nutritional applications.

## 1. Introduction

Aging is a predestined process of the human lifecycle, which is usually accompanied by a gradual decline in physiological, neurocognitive, and metabolic functions. The cumulative effect of these reduced bodily functions weakens the immune system and increases the susceptibility of the aged host to various cardiometabolic, neurodegenerative, and lifestyle disorders. Emerging evidence is now pointing toward the crucial role of gut microbiome homeostasis in host aging-associated health, implicating the role of abnormal microbiome perturbations (‘dysbiosis’), induced by factors such as increased medications and inadequate nutrition with age, in exacerbating the pathophysiology of aging-linked disorders (1, 2). Besides, increased consumption of western-style high-sugar, high-fat and low-fiber diets during old age further aggravates the ill effects of gut dysbiosis-linked aging-associated ailments (3). Earlier studies by our group further corroborated the link between the severity of gut dysbiosis with consumption of a high-fat diet (HFD) in older non-human primates and the amelioration of perturbed gut into a healthier microbiome upon the intervention of high fiber diet, thus underscoring the incorporation of fiber-rich diet in later life (4). As our gut microbiome coevolves with our aging, strategic fiber-rich dietary interventions aimed at maintaining and fostering a robust, diverse and healthier gut microbial ecosystem in the elderly could indirectly prevent/offset the progression of aging-associated diseases thereby potentially reducing the economic burden associated with their treatment costs.

Dietary pulses are an inexpensive, sustainable and valuable source of nutrient-dense, health-promoting foods composed of both high-quality proteins and dietary fibers, which could serve as a perfect-food choice for the elderly in promoting gut and metabolic health (5). In fact, a study, ‘*Foods Habits in Later Life*’ strongly predicted survival among elderly consuming pulses (6). Among many pulse varieties grown worldwide, chickpea, dry peas, lentils and dry beans, including black-eyed peas, pinto beans and kidney beans, are some of the most widely consumed pulses in the United States (7). Collectively, the consumption of whole pulses has been shown to attenuate the risk of chronic diseases by reducing the levels of post-prandial blood glucose, plasma levels of low-density lipoprotein cholesterol, and blood pressure; however, the underlying beneficial mechanisms of whole pulses and/or their constituents on host physiology and gut microbiome in aging milieus remain largely unexplored (8, 9). One such pulse-derived dietary fiber worth exploring is the resistant starch (RS), which remains indigestible in the upper gastrointestinal tract and is finally metabolized by intestinal microbes in the large bowel, thereby promoting gut and metabolic health (10). Apart from exerting health benefits, pulse-derived starches could be a suitable ingredient for functional food formulations, as studied earlier by our group (11). The beneficial effects of RS extracted from other sources, such as acorn and sago plants, in improving physiological responses such as hyperglycemia and insulin sensitivity, reducing gut hyperpermeability and systemic inflammation, and modulating gut microbiome with the production of short-chain fatty acids (SCFAs) had been demonstrated earlier (12). Likewise, the physiologic and gut health effects of RS derived from cereal and tubers have been widely studied, but studies pertaining to pulses-derived RS have only started evolving recently (13–15). Furthermore, majority of these studies utilize *in-vitro* fecal fermentation models, without much extrapolation in *in-vivo* animal models. More importantly, the mechanistic understanding of these pulses-derived RS among aging population is lacking despite extensive use of dietary pulses as staple foods worldwide.

Although human and mouse gut microbiotas share a 90% resemblance at the phyla level, both possess dissimilarities in terms of makeup and abundance of microbial signatures (16). Keeping in view the emerging role of human intestinal microbiome in host physiologic homeostasis, immunity, energy metabolism, and several disease pathophysiologies, the current study was performed using a ‘humanized’ mouse model of aging, i.e., the older mice carrying the healthy human gut microbiome, with the overarching aim to explore the prebiotic attributes of RS purified from four of the most widely consumed pulses (chickpea, black-eyed pea, pinto bean, and lentil) in modulating the gut microbiome, physiological and intestinal health. The insights from this novel study facilitate our understanding of developing nutraceuticals from natural sources to support the health of our ever-growing elderly population. On a broader perspective, this study will facilitate in addressing the nutritional shortcoming of dietary fibers in the US as the consumption of fibers in the country is less than 10% of recommended intake (17).

## 2. Materials and methods

### 2.1. Extraction and preparation of resistant starches from pulses

Chickpeas, pinto beans, black-eyed peas and lentils were batch-ordered and purchased from the commercial vendor^1^ for product consistency and batch-tracking. Isolation and purification of starch from pulse seeds were performed accordingly to our previously described method (11). The recovered starch was dried in an oven (45°C, 24 h) and stored under nitrogen at 4°C. The characteristics of recovered pulse starch are shown in Supplementary Table S1. RS production from purified starches was performed *via* simulation gastric digestion as described previously by Tuncil et al. (18) with slight modifications. Briefly, starch (12 g) was gelatinized in sodium phosphate buffer (240 mL, pH – 6.9). After cooling to 37°C, 2 mL of salivary amylase (Sigma-Aldrich) was added, followed by incubation for 15 min. Under continuous stirring, the pH of the hydrolyzed starch was adjusted from 6.9 to 2.0 using 6 M HCl. Thereafter, digestion was carried out using sequential steps of enzymes: pepsin (37°C, pH 2.0, 30 min) and 4 mL pancreatin (37°C, pH 6.9, 90 min). The hydrolyzed starch was recovered and dialyzed (6–8 kDa, 36 h) and the remaining undigested starch was freeze dried for 72 h.

### 2.2. Animal studies

**Figure 1:**
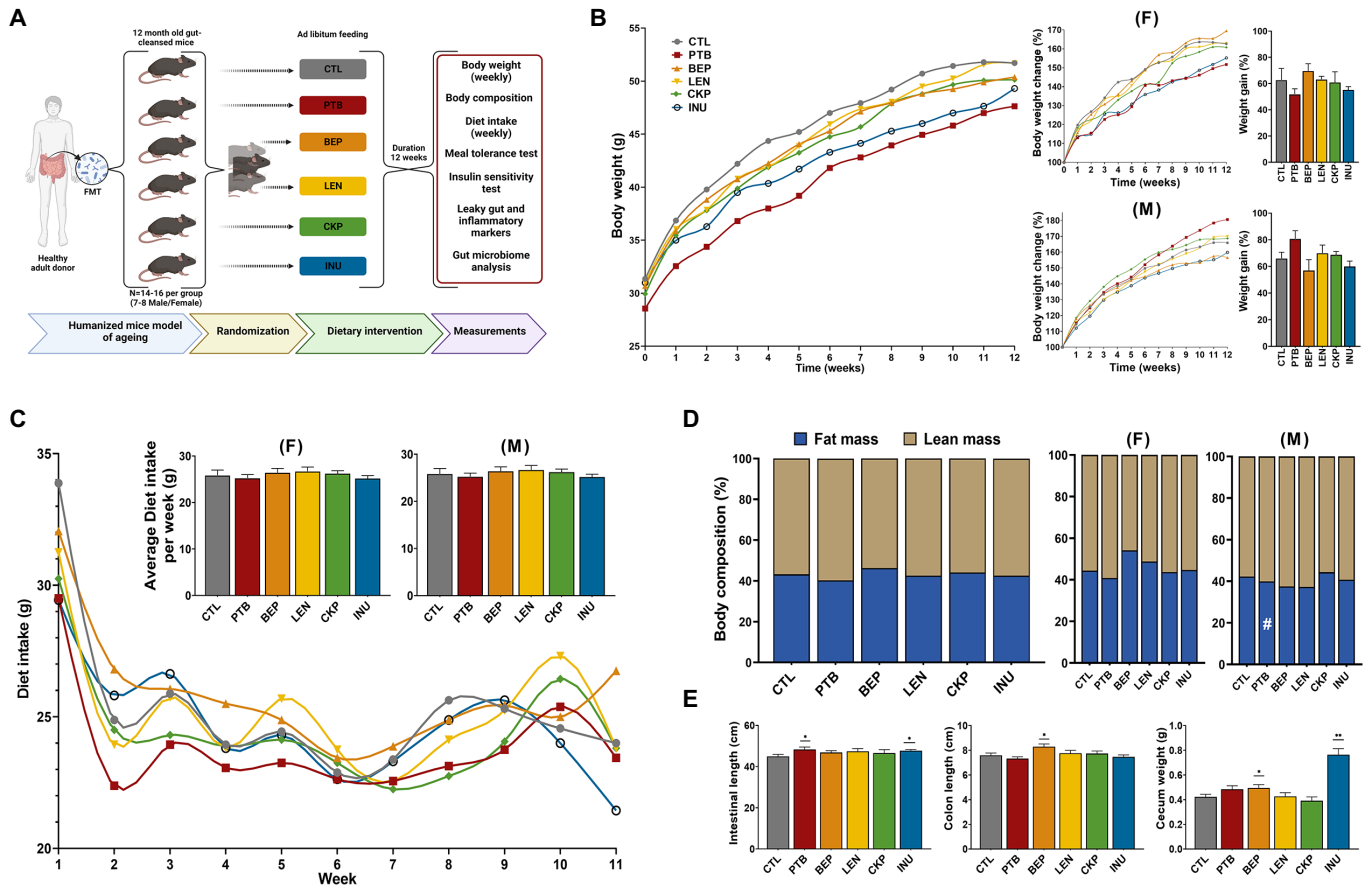
Effects of resistant starches from dietary pulses on bodyweight, diet intake, body-composition, total gastrointestinal length, colon length and cecum weight in mice. **(A)** Experimental Design layout. **(B)** Overall trend depicting weekly change in bodyweight per group along with sex-specific changes in bodyweight (%) and net weight gain (%). **(C)** Overall trend depicting weekly diet intake per group along with sex-specific average diet intake per week per group. **(D)** Overall and sex-specific alterations in body-composition (%) evaluated in terms of total fat and lean mass per group. **(E)** Comparison of total gastrointestinal length, colon length and cecum weight of prebiotics with control. PTB, Pinto beans; BEP, black-eyed peas; LEN, Lentils; CKP, chickpeas; INU, inulin; CTL, control high-fat diet group. ^#^*p* < 0.1, *p < 0.05, **p < 0.01.

The overall layout of the experimental design is summarized in Figure 1A. Briefly, C57BL/6 J mice were purchased at ~55 weeks of age and were allowed to acclimate in the new vivarium for 2 weeks. Then, mice were subjected to our previously validated gut depletion and cleansing procedure to eradicate the gut microbiota (12, 19). Briefly, the gut-cleansing procedure included a 4-day microbial-depletion treatment with a broad-spectrum antibiotic cocktail [containing ampicillin (1 g), Metronidazole (7–11) (1 g), Neomycin (1 g), and Vancomycin (0.5 g) per liter] in drinking water (sweetened with 2% Medi-Drop Sucralose for palatability; Clear H_2_O, Westbrook, ME), followed by bowel cleansing through 4-doses of oral gavage with polyethylene glycol (200 μL per dose; 425 g/L) at 20-min interval on the last day of antibiotic regimen to empty the intestine, wash out the antibiotics remaining in the intestine, and decrease the bacterial load. This regimen depletes more than 95% bacteria from the gut (12, 20, 21). Mice were fasted for 4 h before polyethylene cleansing. Because mice are coprophagous, the cages and bedding materials were renewed each day during this regimen. Then, mice were transplanted with a pool of five older (3 M, 2F; age 50–55 year) subjects’ microbiota (collected recently in our lab; IRB#00002132). To perform microbiota transfer, fecal contents from the five human donors were pooled and dissolved in sterile PBS (100 mg/mL; pH 7.4; pre-reduced in an anaerobic incubator), reconstituted in an anaerobic chamber, and gavaged (200 μL/mice/day) for 4 consecutive days (first FMT given right after 1 h after last polyethylene glycol treatment). For the next one week, the mice were allowed to rest for microbiota stabilization. After one-week, now at 60-weeks of age (matching to middle-aged 50–55 years old humans), the mice were randomly divided into five groups (*n* = 14–16/group; 7–8 for each sex) and were fed a western-style high-fat diet (HFD; #D21080102, Research Diets Inc., New Brunswick, NJ, United States; formulated based on the USDA’s 2008 Dietary Assessment of Major Food Trends) for 20-weeks. One group acted as control (CTL) and received the western-style HFD, while four experimental groups received CTL diet supplemented (5% w/w) with purified RS from dry peas (Black-eyed pea; BEP), lentils (split lentil; LEN), chickpeas (Garbanzo; CKP), and dry bean (Pinto; PTB), respectively. All diet formulations were isocaloric (see Supplementary Table S2). We have previously shown that these four pulses, out of 18 different pulses, retain a considerable proportion of RS pre- and post-cooking and lead to the production of short-chain fatty acids (SCFAs) production by human gut microbiota *in-vitro* (11, 22). One group received a widely studied fiber inulin (5% w/w; Biosynth) as a positive control. The fortification proportion of 5% w/w and inclusion of inulin as a positive control was based on our previous studies with resistance starches and inulin (12). After the 20-week intervention, mice were subjected to a series of assessments as described in the subsequent sections. Mice were about 80–82 weeks old at this time, approximately equivalent to age 60–65 years in humans. Body weight and diet intake were measured weekly. At study endpoint, the mice were anesthetized with isoflurane and euthanized. Cecum weight, liver weight and total gastrointestinal length were measured. Tissues were collected in liquid nitrogen and stored immediately at −80°C for further analysis. All the animal studies and protocols were approved by the Florida State University’s Institutional Animal Care and Use Committee (Protocol #202100008).

### 2.3. Body composition

Total body composition in terms of lean mass versus fat mass (and total water) at the study endpoint was determined in live mice by using the EchoMRI-130 Body Composition Analyzer (EchoMRI, MRI that Counts, Houston, TX).

### 2.4. Gut microbiome analysis

The gut microbiome will be measured as per our previously described methods (4, 12, 23–27). Briefly, genomic DNA from 200 mg of the fecal specimen was extracted using the QIAmp PowerFecal Pro DNA Kit (Qiagen) according to the manufacturer’s instructions. Universal primers 515F (barcoded) and 806R were used to amplify the hypervariable V4 region of the bacterial 16S rRNA gene in accordance with the Earth Microbiome Project benchmark protocol^2^. The resulting amplicons were purified *via* AMPure® magnetic purification beads (Agencourt) followed by quantification through Qubit-4 fluorimeter (InVitrogen), and the final amplicon library was generated as per methods disclosed elsewhere. Equal molar concentrations of the library were pooled and sequenced for paired-end (2 × 300bp) sequencing using an Illumina MiSeq sequencer (using Miseq reagent kit v3; Illumina Inc., San Diego, United States). QIIME2 (ver. 2–2022.8) was used for microbiome bioinformatics analysis (28). Raw sequence demultiplexing and filtering on quality was performed with the q2-demux plugin, followed by trimming and denoising through DADA2 (29). All identified amplicon sequence variants (ASVs) were aligned with the MAFFT (30). ASVs were assigned with a naïve Bayes taxonomy classifier developed for the sklearn classifier against the pre-built from the 99% SILVA 138 database (31, 32).

### 2.5. Meal tolerance test and insulin tolerance test

These assessments were measured as per our previously described methods (4, 12, 33). Briefly, the meal tolerance test (MTT) was performed by oral gavage of 200 μL mixed meal (570 mg Ensure Plus, 30 mg dextrose, and 1.8 mL water) to mice fasted for 6 h. For the insulin tolerance test (ITT), mice fasted for 4 h and were given 0.75 U/kg body weight of insulin (Humulin) intra-peritoneally. Blood glucose reading for both tests was measured at 0 min (before administration), and 15, 30, 60, and 120 min (after administration) using AccuCheck glucometer kit.

### 2.6. *In-vivo* gut permeability assay

Gut permeability was measured per our previously described method (4, 12, 23). Briefly, mice were deprived of food for 4 hours, after which the mice were administered with 60 mg/100 g body weight of fluorescein isothiocyanate (FITC) dextran (4 kDa) solution *via* oral gavage. Two hours after FITC gavage (still on fasting), about 50 μL of blood was collected in a heparinized capillary tube by using a tail vein cut. The concentration of the FITC-dextran was determined in the serum using fluorescence spectroscopy at 530 nm and excitation at 485 nm using a plate reader.

### 2.7. Gene expression analysis for leaky gut and inflammatory markers

Total RNA from the snap-frozen intestinal tissues (colon and ileum) were isolated using RNeasy kit (Qiagen), followed by reverse transcription using the High-capacity cDNA reverse transcription kit (ThermoFisher). The mRNA expression of tight-junction proteins, including claudin-1 (CLDN1), claudin-4 (CLDN4), zonulin-1 (ZO1), zonulin-2 (ZO2), occludin (OCCL) and JAM3 gene; and inflammatory markers including interleukins (IL1β, IL10, and IL17A) and tumor necrosis factor-alpha (TNF-α) were quantified using real-time PCR (QuantStudio3, Applied Biosystems) with primers listed in Supplementary Table S3. The 18S gene was used as an internal housekeeping control. The results were expressed as ddCt method by normalizing against the 18S expression of the control group, as per our earlier report (12, 23).

### 2.8. Statistical analysis

All the values throughout the manuscript are expressed as mean ± standard error of the mean (SEM). Statistical significance between groups was assessed using a two-tailed unpaired *t*-test using Welch’s correction in the GraphPad Prism version 9.4.1. Microbiome analyzes were executed using ‘R’ or ‘Python’ packages. The microbiome diversity indices (α- and β-diversity), as well as microbiome composition in terms of major phyla, families and genera, were compared between control and different treatment groups. Non-parametric Kruskal-Wallis test (34) and PERMANOVA (35) with 999 random permutations were used to identify the significant differences in microbial diversity and structure. All *p*-values involving multiple testings were FDR-adjusted. Supervised classification was performed in the q2-sample-classifier plugin *via* nested stratified 5-fold cross-validation with Random Forest (36) classifier grown with 2,000 trees. The metagenomic functional activities were predcted using the open source bioinformatics tool PICRUSt2 (Phylogenetic Investigation of Communities by Reconstruction of Unobserved States) (37) and output were annotated with KEGG Brite descriptions (38). The sequences were uploaded to PICRUSt2 and were analyzed for the prediction of functional genes of the classified members of the gut microbiota. Subsequently, the inferred gene families were annotated against KEGG (Kyoto encyclopedia of genes and genomes) orthologs (Kos) and then collapsed into KEGG pathways to generate the functional pathway. The functions were finally categorized and compared at levels 2 and 3 as per the methods described elsewhere (37). The linear discriminant analysis (LDA) effect size (LEfSe) (39) was used to identify the difference in bacterial taxa and predicted functions between groups. Additionally, STAMP v 2.1.3 software (40) and Spearman’s correlation were used to detect the group-specific taxa. InteractiVenn (41) was used to illustrate the Venn diagram. Unless otherwise specified, statistical significance was tested at *p* < 0.05.

## 3. Results

### 3.1. Effects of dietary pulses-derived resistant starches on physiological parameters

The overall effect of RS on body-weight, diet intake, body-composition, intestinal/colon length, and cecum weight are summarized in Figures 1B–E. There was no marked impact of different RS on the body-weight of either male or female mice. Although non-significant, the INU group demonstrated less weight gain overall, whereas PTB and BEP groups showed restricted weight gain in females and males, respectively (Figure 1B). There was no difference in the food intake in either gender (Figure 1C). Further, no notable differences were observed between fat and lean mass of control and experimental groups; however, male mice from PTB group exhibited marginally lower fat mass gain (*p* = 0.0781) compared to CTL (Figure 1C). Overall, the intestinal length was significantly longer for PTB and INU groups whereas an extension in colon length was observed for BEP group (Figure 1E) mainly in males (Supplementary Figure S1). Moreover, BEP (*p* < 0.05) and INU (*p* < 0.0001) had significantly heavier cecum than CTL. INU group-associated increase in cecum weight was consistent in both sexes (Supplementary Figure S1). No overall difference in liver weight was observed, but PTB group females demonstrated low liver weight (*p* < 0.05) compared to CTL (Supplementary Figure S1). Altogether, no single group yielded consistent results for these parameters, although some overlapping effects were observed for INU group in both sexes, PTB in females, and BEP in males.

### 3.2. Resistant starches from different beans and pulses differently modulate the gut microbiome

**Figure 2:**
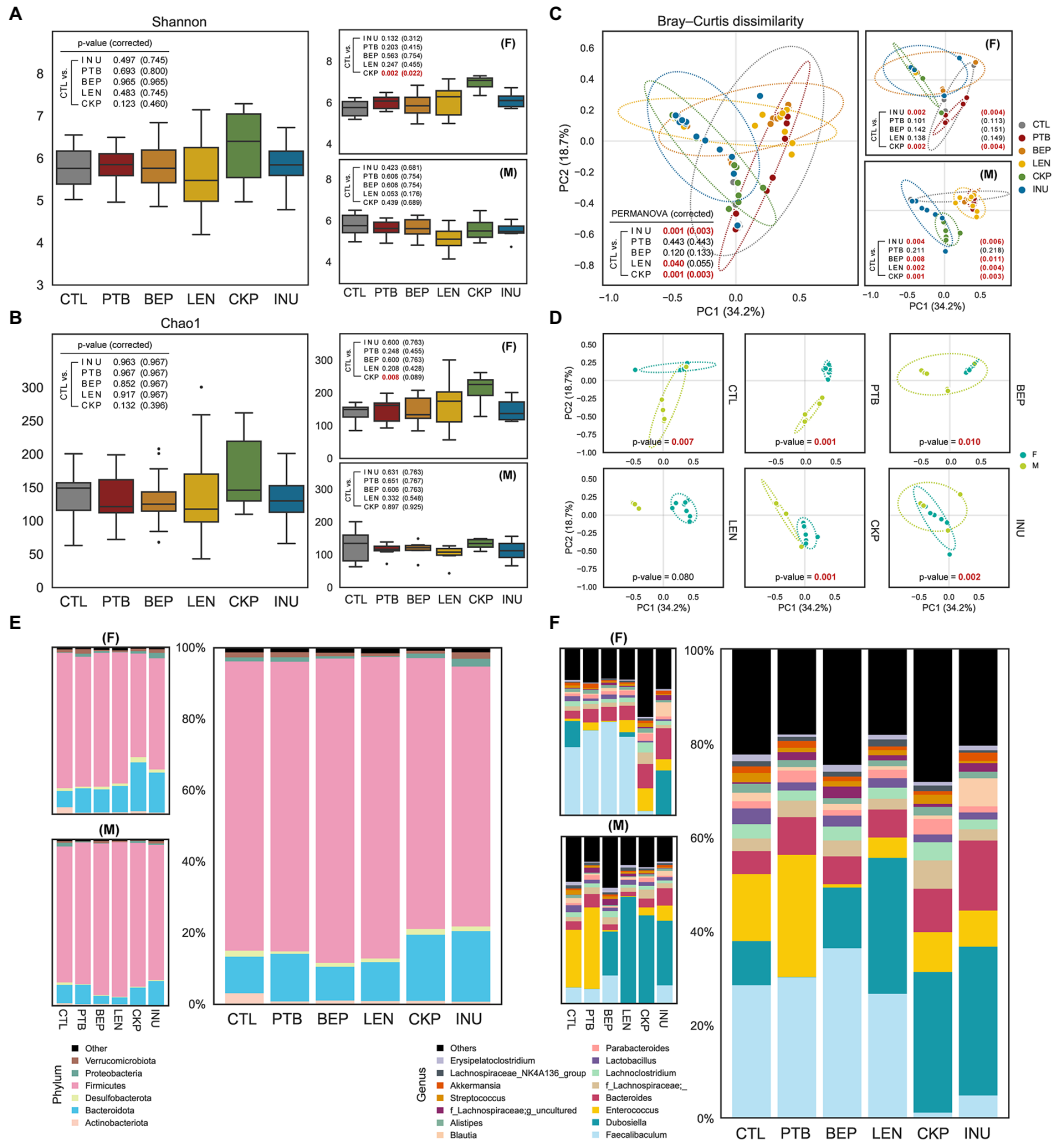
Effect of resistant starches from dietary pulses on gut microbiome diversity and taxonomic hierarchies. Alpha-diversity of microbiome of each group determined using **(A)** Shannon index and **(B)** Chao1 index. **(C)** PCoA analysis representing β-diversity of microbiome of each group combined or male and female groups. **(D)** Sex-specific differences in microbiome composition for each group; covariance ellipses are displayed and bounds (dotted lines) of each group with two standard deviations (2σ) in each direction from the group mean. Relative abundance of gut microbiome in male and female mice or all combined at **(E)** phylum level and **(F)** genus level. PTB, Pinto beans; BEP, black-eyed peas; LEN, Lentils; CKP, chickpeas; INU, inulin; CTL, control high-fat diet group. *p < 0.05.

The overall and sex-specific impact of RS on the α-diversity of the gut microbial community was depicted using Shannon and Chao1 index, as depicted in Figures 2A,B. No significant differences in α-diversity were observed overall, but CKP significantly enhanced α-diversity in females compared to CTL as revealed by Shannon index (*P*_FDR_ = 0.022). However, the β-diversity revealed notable differences in the microbial structure between CTL and RS-treated groups. Both INU and CKP caused significant structural changes in both sexes, whereas BEP (*P*_FDR_ = 0.011) and LEN (*P*_FDR_ = 0.004) induced differences only in males (Figure 2C). Besides, sex-specific effects of different RS on β-diversity were observed, as presented in Figure 2D. Compared to the CTL group, the overall proportion of major bacterial phyla including Firmicutes and Bacteroidota did not differ between groups, but the LEN group harbored a significantly higher proportion of Firmicutes (male-driven) while Bacteroidota (female-driven) was more abundant in the CKP and INU groups (Figure 2E). Furthermore, phyla Proteobacteria and Actinobacteria decreased by LEN and INU intake, respectively. Further analysis at the bacterial genus level revealed *Faecalibaculum* (female-dominated), *Dubosiella* (male-dominated), *Enterococcus*, and *Bacteriodes* as dominant genera accounting for 50–60% of total abundance across different groups (Figure 2F). Overall, LEN had a maximum abundance of former two genera. Interestingly, *Faecalibaculum* decreased in CKP and INU. *Enterococcus* was found in more abundance in PTB but was reduced in other groups compared to CTL. INU had the highest rise for *Bacteriodes*. A detailed description of hierarchical clustering at bacterial phylum, family, and genus levels for individual samples is presented in Supplementary Figure S2.

**Figure 3:**
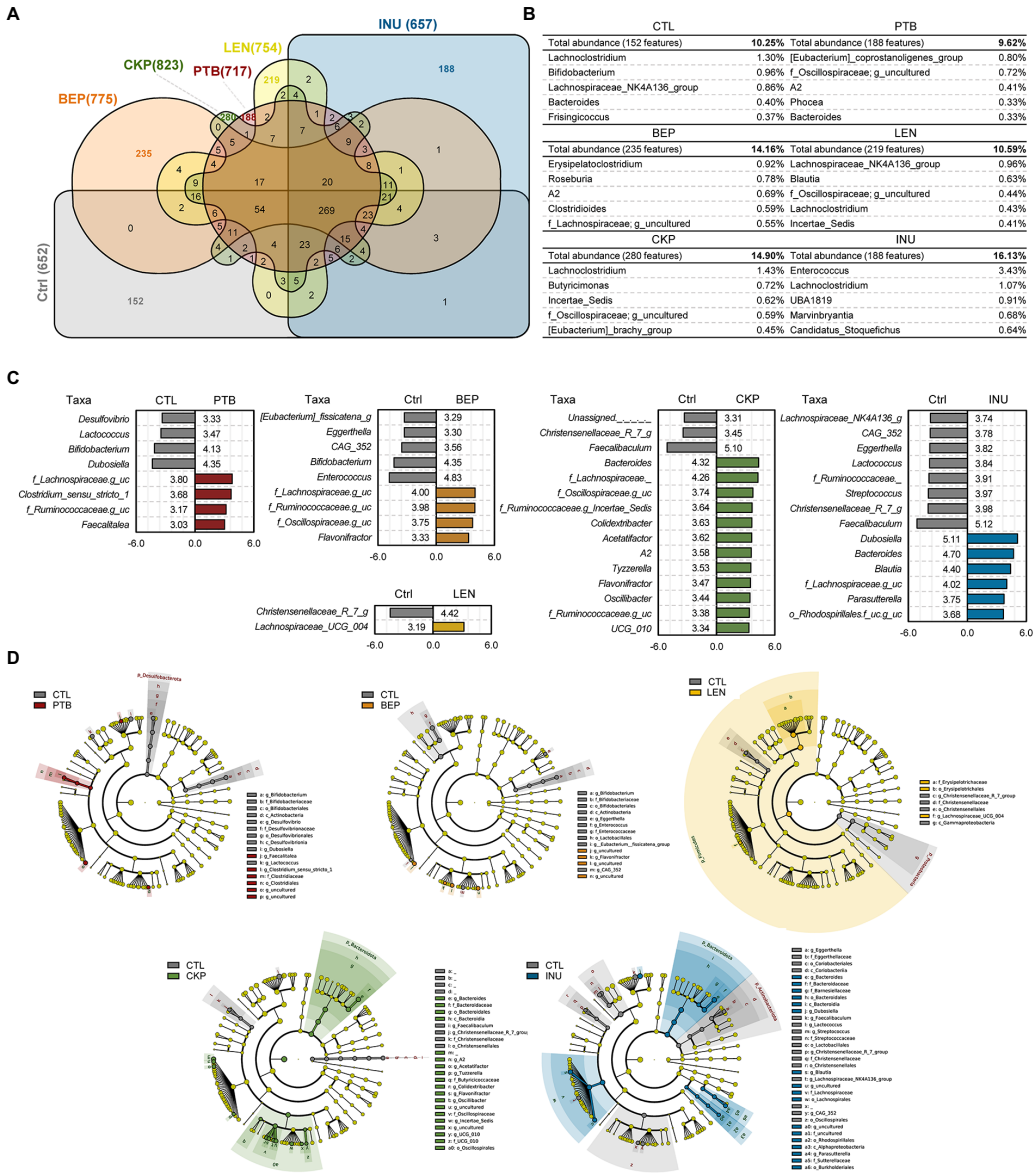
Comparison of unique microbiome features and discriminatory taxa between prebiotics (resistant starches and inulin) versus control groups. **(A)** Venn diagram illustrating unique and shared features (ASVs) among different groups. **(B)** Unique features expanded to display top 10 genera with relative abundance of unique features of each group. **(C)** Bacterial composition differences between groups analyzed with Linear discrimination analysis (LDA) effect size (LefSe) algorithm; *x*-axis: LDA score. **(D)** LEfSe cladogram depicting differentially abundant taxa between groups. PTB, Pinto beans; BEP, black-eyed peas; LEN, Lentils; CKP, chickpeas; INU, inulin; CTL, control high-fat diet group.

Among total 1,898 amplicon sequence variants (ASVs) obtained, the largest number of unique ASVs (280, 14.8%) were identified in the CKP group, followed by BEP (235, 14.16%) and LEN (219, 10.59%) (Figures 3A,B). The identification of characteristic bacterial taxa changes at genus and various taxonomic levels were further visualized using the linear discriminant analysis (LDA) scores and LDA effect size (LEfSe)-based cladogram analyzes, respectively (Figures 3C,D). PTB group demonstrated a significantly higher proportion of *Lachnospiraceae*, *Clostridium sensu stricto-1*, *f_Ruminococcaceae* and *Faecalitalea* while having a lower proportion of *Desulfovibrio*, *Lactococcus*, *Bifidobacterium* and *Dubosiella*. In BEP group, *Lachnospiraceae*, *Ruminococcaceae*, *Oscillospiraceae*, and *Flavonifractor* increased, while *Eubacterium fissicatena group*, *Eggerthella*, *CAG_352*, *Bifidobacterium* and *Enterococcus* decreased. The LEN group showed relatively least significant difference compared to the CTL group with *Christensenellaceae R-7 group* deceased and *Lachnospiraceae UCG-004* increased compared to CTL. CKP exhibited an increase in 12 genus-specific taxa compared to CTL, which included *Bacteroides*, *Lachnospiraceae*, *Oscillospiraceae*, *Ruminococcaceae-g_Incertae_Sedis*, *Colidextribacter*, besides several other taxa, whereas assigned taxa including *Christensenellaceae R-7 group* and *Faecalibaculum* decreased significantly. For INU group, *Dubosiella*, *Bacteriodes*, *Blautia* and *Lachnospiraceae* were among the selected taxa that increased and *Ruminococcaceae*, *Streptococcus*, *Christensenellaceae_R_7_g*, and *Faecalibaculum* were among the decreased ones.

**Figure 4:**
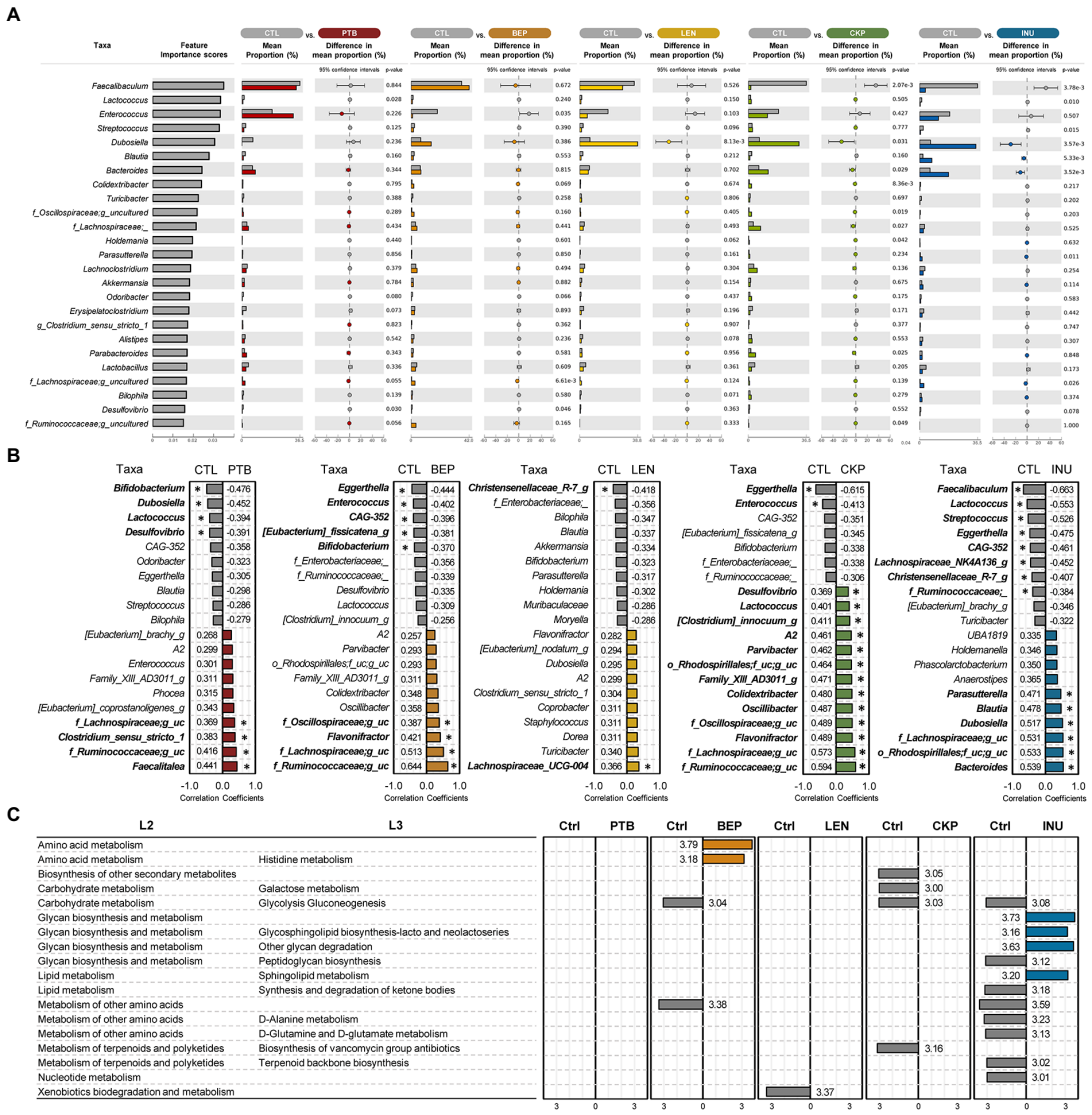
Comparison of predictive microbial genera, functional profiles and correlation analysis between resistant starches and control groups. **(A)** The top 25 most strongly predictive genera ordered by relative importance score used to assess the contribution to classifier accuracy, and extended error bar plots with corrected value of *p* (Welch’s two-sided *t*-test) for each taxon. **(B)** Correlation analysis for top 20 genera. **(C)** Differences in predictive KEGG functional capabilities using the LEfSe (LDA > 3.0) analysis. PTB, Pinto beans; BEP, black-eyed peas; LEN, Lentils; CKP, chickpeas; INU, inulin; CTL, control high-fat diet group. *p < 0.05.

To identify discriminatory taxa associated with each RS, we applied a supervised machine learning-based random forest model trained with genus-level abundance. The model demonstrated a high classification accuracy for each RS (AUC = 0.70–0.95) and the overall accuracy of prediction was 60.4% (Supplementary Figures S3A,B). Among the 25 most predictive genera influenced by RS, 16 genera showed a significant difference between CTL versus at least one of four RS groups (Figure 4A). The prominent predictive genera included *Faecalibaculum*, *Lactococcus*, *Enterococcus*, *Streptococcus*, *Dubosiella*, *Blautia* and *Bacteroides*. The presence of these top genera, along with *Odoribacter*, *Desulfovibrio*, *Lactobacillus*, *Alistipes* and *Erysipelatoclostridium* was predominant in CTL group. Except for PTB, *Dubosiella* was more prevalent in other groups compared to CTL. *Bacteroides* predicted more strongly towards CKP and INU groups.

*Colidextribacter*, *Holdemania* and *Lachnospiraceae;g_* were significantly associated with CKP, while *Parasutterella* and *Blautia* were linked with INU group. Correlation analyzes between control and treatment groups for the top 20 genera are summarized in Figure 4B. The genera exhibiting significantly positive and negative correlations among different groups versus control were nearly identical to those found significantly different by LEfSe analyzes, except for CKP, which showed additional insights for genera associated negatively (*Eggerthella* and *Enterococcus*) and positively (*Desulfovibrio*, *Lactococcus*, *Parvibacter*, besides others) correlated. In general, positively (*Lachnospiraceae;g_uncultured*, *Ruminococcaceae;g_uncultured*, *Flavonifractor* and *A2*) and negatively correlated genera (*Bifidobacterium*, *Eggerthella*, *CAG-532*, *Enterobacteriaceae*;g) shared common features in at least three of RS groups compared to CTL. KEGG-based functional analysis annotated these microbiome changes to a total of 12 differential metabolic pathways when compared to CTL (Figure 4). Overall, pathways pertaining to amino acid, vitamins, carbohydrate, glycan, and lipid metabolism were upregulated prolifically in all RS groups (Supplementary Figure S4). Pathways specific to KEGG level-3 that upregulated in specific treatment groups included histidine metabolism (BEP); and sphingolipid metabolism and glycan synthesis (INU). On the other side, downregulated pathways were linked with glycolysis gluconeogenesis (BEP, CKP, INU); xenobiotics degradation (LEN); galactose metabolism (CKP); biosynthesis of peptidoglycan and terpenoids (INU); metabolism of D-alanine, D-glutamine, and nucleotides (INU).

### 3.3. Resistant starches from different dietary pulses restrict elevation in post-prandial blood glucose levels independent of insulin sensitivity

**Figure 5:**
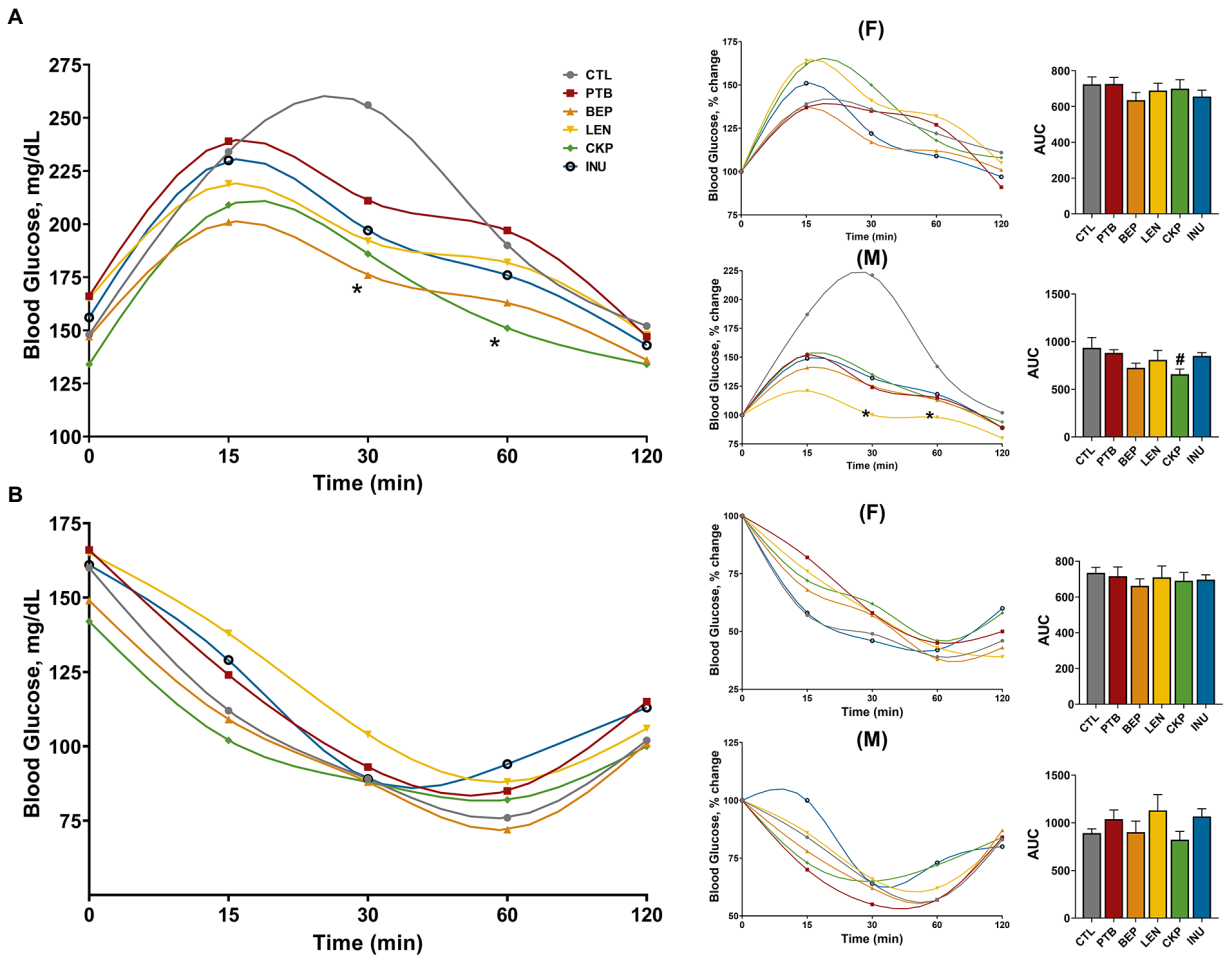
Effect of resistant starches from dietary pulses on post-prandial blood glucose levels and insulin sensitivity. **(A)** Meal tolerance test and **(B)** Insulin tolerance test in combined or separately in males and females along with sex-specific percent change in blood glucose levels and area under curve (AUC). PTB, Pinto beans; BEP, black-eyed peas; LEN, Lentils; CKP, chickpeas; INU, inulin; CTL, control high-fat diet group. ^#^*p* < 0.1, *p < 0.05.

The overall changes in blood glucose levels, along with sex-specific percent changes in glucose and area under the curve (AUC) during the oral meal and intra-peritoneal insulin administration, are depicted in Figures 5A,B. Overall, BEP and CKP groups yielded lower blood glucose after 30 and 60 min of meal administration, respectively (Figure 5A). Besides, males from the CKP group presented the lowest AUC (*p* = 0.0782) for blood glucose. Although no significant variations in meal tolerance were observed for females, LEN group in males rendered a relatively lower yet stable trend towards change in post-prandial glucose levels compared to CTL. Regarding insulin sensitivity, no significant differences were observed among control and treatment groups in male or female mice (Figure 5B), although a marginal decrease in AUC for CKP group males might suggest improved insulin sensitivity. Overall, CKP and LEN groups offered protection to some extent against post-prandial glucose levels in males.

### 3.4. Resistant starches from different dietary pulses ameliorate gut permeability and leaky gut markers

**Figure 6:**
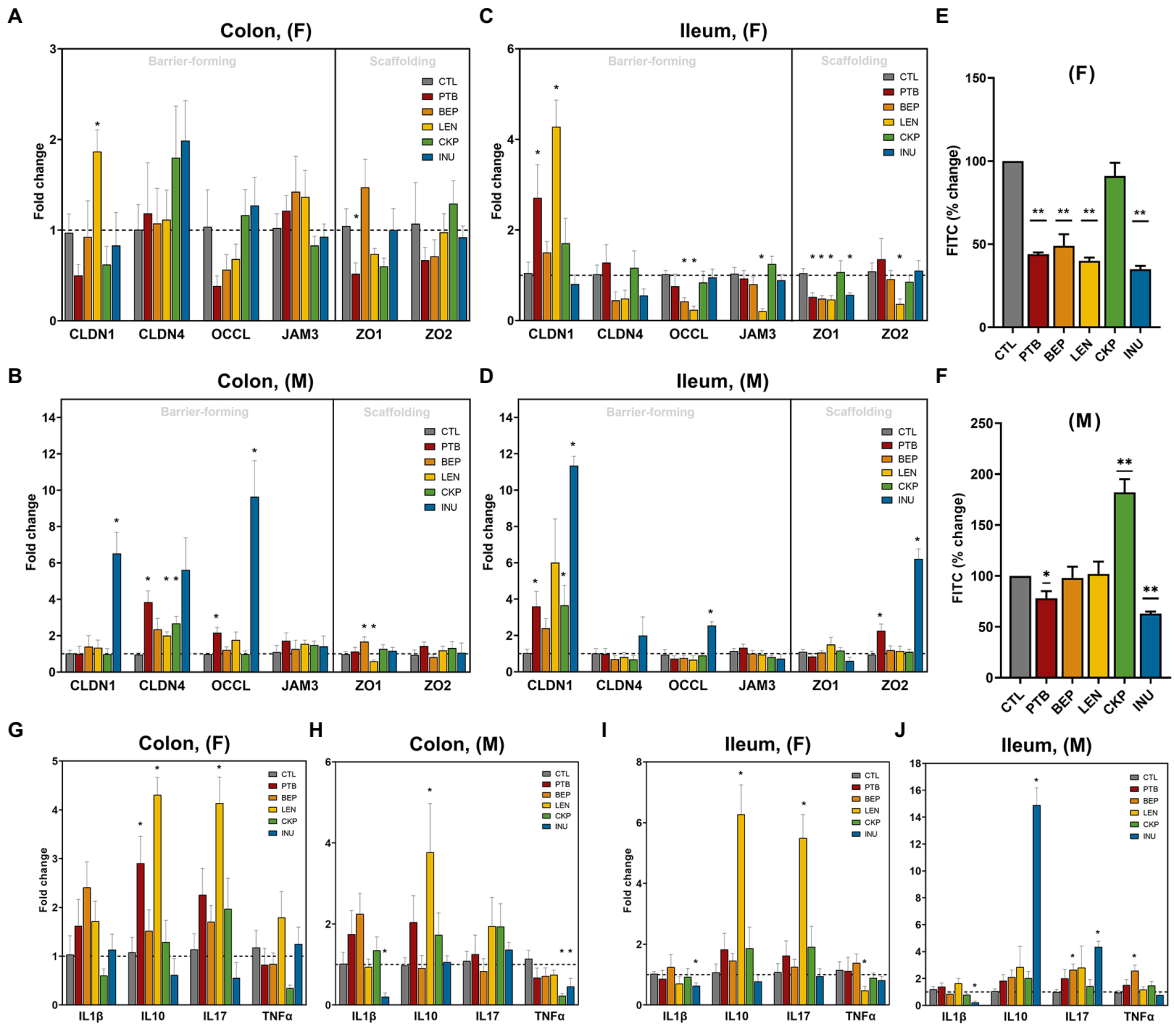
Effect of resistant starches from dietary pulses on the markers of gut permeability, intestinal integrity, and inflammation. Gene expression of tight-junction proteins *viz.*, claudins (CLDN-1 and CLDN-4), zonulins (ZO1- and ZO-2), occludin (OCCL), and JAM3 in **(A)** females’ colon, **(B)** males’ colon, **(C)** females’ ileum and **(D)** males’ ileum. Gut permeability (in terms of the diffusion of 4 kDa FITC dextran from gut to blood circulation) in **(E)** females and **(F)** males. Gene expression of inflammatory markers including interleukins (IL1β, IL10, and IL17) and tumor necrosis factor-alpha (TNFα) in **(G)** females’ colon, **(H)** males’ colon, **(I)** females’ ileum and **(J)** males’ ileum. PTB, Pinto beans; BEP, black-eyed peas; LEN, Lentils; CKP, chickpeas; INU, inulin; CTL, control high-fat diet group. *p < 0.05, **p < 0.01.

The influence of RS on intestinal epithelial layer integrity in both males and females was examined in terms of the mRNA expression of different barrier-forming and scaffolding genes in ileum and colon tissues (Figures 6A–D). Among females, LEN group increased CLDN-1 expression in both tissues while expression of OCCL and JAM3 decreased in the ileum with no major impact on the colon (Figures 6A,C). The scaffolding proteins (ZO1 and ZO2) mostly remained unaffected in the colon of females, whereas all groups except CKP downregulated ZO1 expression in the ileum. Moreover, PTB group enhanced CLDN-1 expression in the ileal tissues of both male and female mice (Figures 6C,D). INU group exerted male-specific enhanced expression of CLDN-1 and OCCL in the colon and ileum (Figures 6B,D). CLDN-4 was upregulated by PTB, LEN and CKP groups in the males’ colon (Figure 6B). While ZO2 exhibited no significant regulation in males’ colon but found to increase in the ileum by PTB and INU groups (Figure 6D). Furthermore, we also measured gut leakiness by gut permeability assay using oral FITC-dextran administration in fasted mice, the findings of which are depicted in Figures 6E,F. Overall, PTB and INU reduced gut leakiness in both sexes compared to CTL. BEP and LEN had significantly improved gut permeability only in females (Figure 6E). Unexpectedly, CKP group males had increased gut permeability, with no major reduction observed in females. This finding provides a divergent result compared to leaky gut expression analysis as the positive effect CKP on increased expression of barrier-forming genes (CLDN1 and CLDN4) in males was observed.

### 3.5. Resistant starches from different dietary pulses differentially influence inflammatory markers in males and females

The overall gender-specific impact of RS in modulating the expression of pro-inflammatory (IL1β, IL17A, and TNFα) and anti-inflammatory (IL10) cytokines are presented in Figures 6G–J. In females, IL10 and IL17A increased consistently in the colon and ileum upon LEN treatment, while PTB supplementation increased IL10 in the colon (Figures 6G,I). In males, IL10 was increased by LEN in the colon and INU in the ileum (Figures 6H,J). In females, LEN significantly downregulated TNFα expression in the ileum along with its non-significant reduction in the colon by CKP (Figures 6G,I). For males, a decrease in TNFα was observed in the colon by CKP and INU groups, while a BEP-mediated increase occurred in the ileum (Figures 6H,J). The decline in IL1β expression by INU group was evident in the ileum of both males and females and in the colon of males (Figures 6H–J). Altogether, RS supplementation enhanced anti-inflammatory cytokine (IL10) and declined pro-inflammatory (TNFα) response in both sexes. In addition, a positive correlation was observed between IL10 and IL17A cytokines levels, which was strikingly evident for the LEN group.

## 4. Discussion

Dietary modulation of the gut microbiome to ameliorate pathophysiology of various aging-associated non-communicable diseases such as obesity and type-2 diabetes is receiving remarkable attention. Among these modulatory approaches, dietary fibers, including resistant starches, could be a promising ingredient for gut health owing to their ability to safely pass through the upper gastrointestinal tract and act as good substrates for fermentation by colonic microflora. To this end, the current investigation evaluated the prebiotic potential of resistant starches extracted from the different dietary pulses, *viz.* pinto beans, black-eyed peas, lentils, and chickpeas on the gut and metabolic health outcomes in a ‘humanized’ mouse model of aging. RS supplementation within a high-fat diet (HFD) induced distinct sex- and starch-specific alterations in the gut microbiome, post-prandial hyperglycemia, leaky gut, and inflammatory markers in human microbiome-harboring mice. The overall effect of RS supplementation on weight gain did not yield significant differences compared to CTL till 12-weeks. Although the protective effect of RS type-2 (RS2) on high-fat diet has previously been demonstrated earlier (42), weight reduction in some studies was not evident (43, 44). Moreover, a recent study reported detectable weight reduction after 12-weeks of intervention, suggesting delayed therapeutic effects of RS2 (3). Also, the anti-obesity effects of RS are dose-dependent with diets containing at least 8% RS exhibiting reduced adiposity irrespective of phenotype (45). We also observed statistically insignificant but noticebale sex-specific restricted weight gain for PTB and BEP groups. Sex-specific differences can also affect the trajectory of weight gain as studied previously by feeding RS (a retrograded amylose starch) supplemented diet to human microbiome-harboring rats (46). The terminal bodyweight of male rats was increased while that of females either remain unaffected or decreased after RS consumption. RS-mediated increases in cecum weight have been reported earlier (43), which may reflect the enhanced fermentative ability of gut microbes guided by dietary fibers (47). RS-enriched diets have been shown to promote gastrointestinal length, which, especially for colon, may also reflect on the proportion of RS entering the colon (48). Besides, an extension in colon length is also considered as a useful indicator of reduced intestinal inflammation associated with improved barrier function, mucosal repair, and enterocyte hyperplasia (49).

Compelling evidence has demonstrated the modulating role of RS in the restructuring of gut microbiome composition, which is further dictated by the selective ability of gut microbes to metabolize different RS types (50). In the present study, α- and β-diversity arrays were affected differently by the consumption of RS from different sources, with CKP demonstrating a more pronounced effect overall. Such differences could be ascribed to structural variations in RS linked with different legume cultivars (5). Whole chickpea powder supplemented in an obesogenic diet has previously been reported to yield superior α-diversity compared to other pulse groups (9). Likewise, we noted that different RS groups exhibit differential effects across all taxonomic hierarchies, which further vary by gender. LEN exhibited positive effects at the phylum level by enhancing Firmicutes (predominant in males) and depleting Proteobacteria. These results provide divergent findings as Firmicutes are generally decreased after consumption of whole lentils in a high-fat diet (9), signifying the interactive effects of other lentil components in the diet. Increased abundance of Firmicutes at the expense of Bacteroidetes in individuals receiving RS2 has also been reported previously (51). Many Firmicutes members are associated with butyrate production, whereas Proteobacteria encompass many lipopolysaccharides (LPS)-producing species which may induce gut dysbiosis and the onset of several diseases (3, 52). Besides, Bacteroidota (predominant in females), which increased after CKP and INU treatment, harbors genera having the ability to degrade complex carbohydrates, including RS, to produce acetate and propionate (52). The influence of gender in orchestrating gut microbiome composition has received considerable attention lately as microbiota differences among sexes could contribute to variation in local and systemic inflammation as well as susceptibility to an array of cardiometabolic diseases. Studies have emphasized to consider gender-specific effects while understanding the interactions between gut microbiome and diet (53). Previous study by our group established gender-specific microbiome changes upon yoghurt consumption, wherein consumption patterns in male cohorts were negatively correlated with *Lactilactobacillus sakei*, *Staphylococcus* and *Enterobacteriaceae*, whereas *Lacticaseibacillus casei* found positively correlated with females (54). Sex-specific alterations in the gut community structure of rats receiving prebiotic (oligofructose)-enriched chow have also been documented (55), wherein female rats exhibited predominance of phylum Bacteroidetes while no major changes in fecal community structure of males were observed. This highlights the importance of including both sexes during dietary intervention studies involving animal models to fully elucidate the universality of health benefits imparted by dietary fibers and prebiotics.

The specific effects of RS in increasing the abundance of some reported SCFAs producers, *viz.*, *Faecalibaculum*, *Dubosiella*, and *Bacteriodes* and reducing obesity-associated genera (e.g., *Enterococcus*) corroborate well with recent studies (14, 56). Moreover, *Enterococcus*, which had high relative abundance in CTL group of males, was not suppressed by PTB intervention. However, this consequence exhibited no detectable adverse outcomes in terms of glucose response, inflammation, and gut leakiness for PTB group in males. Even though some species of *Enterococcus* are opportunistic pathogens, many others are known to be beneficial or commensal to the host. For instance, *E. hirae* has been reported to enhance anti-cancer immune responses and improve gut epithelial integrity (57). Other studies have also reported increased *Enterococcus* levels in rats by consumption of high-amylose maize starch and its protective role in reducing bile acid toxemia by prompting the excretion of primary bile acids from the intestine (58).

The *Ruminococcaceae*, *Oscillospiraceae*, and *Lachnospiraceae* families among Firmicutes phylum harbor varied SCFAs-producing genera, with many of their uncultured members observed to be enriched in RS groups as revealed by the LEfSe analysis (59). *Lachnospiraceae_UCG_004*, a genus abundant in the LEN group, has been reported to be depleted in an altered gut microbiome of patients with coronary artery diseases (60). Some of the well-known members of *Oscillospiraceae* (e.g., *Faecalibacterium*) aid in the production of butyrate and have the capacity to metabolize glucuronate (61). Compared to CTL, RS groups significantly downregulated known opportunistic harmful bacteria, including *Desulfovibrio* [PTB], *Eggerthella* [BEP, CKP] and *Enterococcus* [BEP, CKP], which are associated with inflammatory conditions in the gut (62). In addition, various potential pathobionts among several discriminatory taxa identified using a machine-learning approach, such as *Odoribacter*, *Alistipes* and *Erysipelatoclostridium* were found more abundant in CTL group, further highlighting the potential of RS in suppressing potentially harmful gut microbial inhabitants. Previous studies have also demonstrated the regulatory role of RS in inhibiting the growth of *Erysipelatoclostridium*, *Desulfovibrio*, *Alistipes* (aging-associated taxa) and *Odoribacter* (adiposity-linked taxa) in HFD-fed mice (3, 63, 64). In a recent study, the abundance of *Streptococcus* in CTL compared to RS groups was reported, which could be attributed to obesity-induced inflammatory responses (65). *Christensenellaceae R-7* group, which exhibited a negative correlation with LEN and INU groups, has also been reported to be inhibited by high amylose content in RS-enriched diets (66). Members of the *Christensenellaceae* family are associated with reduced adiposity in general and are specialized in protein fermentation (67). Recent studies on [*Eubacterium*]_*fissicatena_g* (negatively correlated with BEP) showed its close association with hypertension (68). However, the same has also been linked to reduced body weight and adiposity (69). Nevertheless, the current knowledge on the clinical importance of these two genera with respect to metabolic disorders is still limited and needs further investigation. Considering the ambiguous role of some genera (e.g., *Enterococcus*, *Eubacterium*) on host metabolism, further species level identification using shotgun metagenomics would provide more information on the potential mechanisms of RS on gut health as a function of species within a particular genus. Although *Bifidobacterium* was negatively correlated with RS groups in the current study, it was not among the top 25 discriminatory taxa associated with each RS by the machine learning algorithm. This is in contrary to the previous studies reporting an increased prevalence of *Bifidobacterium* in rodent and *in-vitro* fermentation models following RS intervention (13, 70). However, the growth of *Bifidobacterium* and *Lactobacillus* may also be affected by the fat content in RS-supplemented diet (71). Nonetheless, the response of gut microbiome to RS fermentation is quite complex, which may be affected by nuanced structural and physicochemical variations in RS derived either from same or different botanical origin (72). Studies have attributed microstructural features of RS such as amylose content, degree of crystallinity (B-type), chain-length and branching density, blocklet type, surface porosity and texture as a principal factors behind enhanced resistance of RS to digestion and its subsequent differential modulation of gut microbiome (73, 74). Depending upon the source, retrogradation process and amylose content of native starches, formation of RS3 with B-type polymorphic form, low surface porosity, having a compact and order blocklet structure along with higher proportion of chains with moderate degree of polymerization (37–100) could act as superior prebiotic for gut and metabolic health, as investigated recently by (75). Even though mining these physicochemical structural variations in RS was not the prime focus of this study, accumulating evidence points out the governing role of fine structural variations of RS in modulating the gut microbiome differently, which may be investigated in future to delve deep into targeted microbiota interventions.

The ASVs of each treatment group retrieved from 16S rRNA sequencing yielded alterations in their metabolic pathways compared to CTL. The upregulation of genes involved in lipid and carbohydrate metabolism by chickpea RS had been demonstrated, which are consistent with our study (65). The underrepresentation of genes involved in carbohydrate (glycolysis/gluconeogenesis and galactose) and amino acid metabolism (D-alanine and D-glutamate) by RS consumption has been previously reported (66). In addition, these studies have reported a positive association of *Alistipes* with former metabolism and a negative association of *Parasutterella* with the latter, which were also evident in our study. The histidine metabolism pathway, which was only affected by BEP seems to play a role in body fat reduction and lower levels of systemic inflammation (76). Upregulation of the sphingolipid signaling pathway further emphasizes diet-induced restructuring of the gut microbiome towards improved metabolic function (77). However, it may be noted that the functional prediction using PICRUSt based on 16 s rRNA gene may not be exhaustive as ASVs are annotated using only about 300 bp amplicon read length and thus may have limited application to predict actual complex functional events. Nevertheless, it provides novel hypotheses and future research direction to gain deeper insights on the functional consequences of RS on gut and host health using comprehensive and inclusive approaches such as whole genome metagenomics and metatranscriptomics.

Intake of dietary fibers, including RS are widely reported to lower post-prandial glucose response, improving insulin sensitivity, and modulate energy metabolism (75). In the current study, the physiological benefits of RS (mainly LEN and CKP) on faster glucose clearance during oral meal administration were mostly observed in males during HFD-feeding. The anti-obesity effects of native (RS2) and retrograded (RS3) lentil starch revealed decreased blood glucose following 6-weeks of HFD-feeding (14). The modulatory role of dietary chickpeas and their RS in controlling post-prandial hyperglycemia, hyperinsulinemia and hyperlipidemia has been reported earlier (65, 78). In line with our study, sex-specific variations in glycemic responses to western-style diet have also been reported in wild-type C57BL/6 mice with males demonstrating a higher response to GTT than females (79). Higher glucose tolerance of females than males could be associated with increased levels of adiponectin and reduced oxidative stress conferred by estrogen (80), which could be a plausible factor explaining no marginal change in glycemic response of females post RS intervention. Insulin sensitivity was also marginally influenced by CKP group in males in current study. Improvement in insulin sensitivity upon RS intervention has earlier been found to be effective for men with no apparent benefit observed in women (81). Apart from gender effects, such RS-specific variation in the glycemic response could be intimately linked to differences in the structural characteristics of different pulse starches such as amylose chain length, granule shape and size, porosity, B-polymorphic content, degree of polymerization and amylose/amylopectin ratio in the starch (82).

Low-grade inflammation and gut hyperpermeability could further fuel the pathogenesis of various metabolic diseases in the elderly (83). We have previously shown how HFD-induced perturbations in the gut microbiome may exacerbate pro-inflammatory signals *via* microbe-associated lipopolysaccharide (LPS) production, thereby stimulating gut leakiness and endotoxemia (12). Overall, the current study showed that the RS supplementation ameliorated intestinal dysfunctions in both sexes; however, the regulatory effects of RS on tight-junction (TJ) and inflammatory genes are sex-specific. The variation in the expression of TJ genes in the intestine of males and females have been reported in previous studies (84) reporting altered expression of multiple TJ genes in the intestine of males and females using zonulin transgenic mouse model and revealing sex-specific altered TJ genes expression with reduction of CLDN15, CLDN5, JAM3, and Myo1C genes only in males’ duodenum while downregulation of CLDN15, CLDN7, and ZO2 in females’ colon. Earlier study using same model demonstrated increased gut permeability in both genders with slightly more pronounced effect on males than females (85). In line with this, we observed reductions in the gut permeability *via* FITC-dextran assay among different RS and INU groups, which could be attributed to increased expression of tight-junction proteins such as CLDN1, CLDN4 and OCCL in the intestinal tissues. Lower expression of claudins is associated with a hyper-permeable mucosal environment and is correlated with the onset of type-1 diabetes (86). Although CKP did not exhibit any lowering effect on gut permeability, it did enhance the expression of some barrier-forming genes (CLDN1 and CLDN4), which could likely be due to the reason that CKP induced highest bacterial diversity with upregulation of many beneficial genera in beside some other selected genera namely, *Tyzzerella* and *Oscillibacter*, which have earlier been associated with obesity and impaired gut permeability (3). Though we have not analyzed fecal butyrate levels, increased gut permeability in males could also be attributed butyrate-dependent faster turnover of intestinal cells *via* colonocyte differentiation and maturation (55). Further experiments focusing on the fecal concentration of SCFAs would provide more evidence on the beneficial effects of RS on host health.

Ample evidence suggests the crucial role of sexual dimorphism in immune responses, with reported differences in basal level of pro-and anti-inflammatory cytokines in males and females (55, 87). High IL-10 and low TNF-α expression, which was more prominent in LEN, CKP and INU groups, respectively, further highlighted their potential for improving aging-associated intestinal immunomodulation. A similar trend in intestinal inflammation, as measured by mRNA expression, by retrograded starch extracted from *Arenga pinnata* has previously been reported in aged mice (88). IL-10 secreted by Tregs confers anti-inflammatory effects by controlling the production of pro-inflammatory cytokines like TNF-α while interacting with dendric cells and macrophages. We find that concomitant to IL-10, IL-17 expression was also enhanced by LEN. Innate production of IL-17 under homeostatic conditions is primarily driven by intestinal γδ T-cells, whereas Th17 cells control the adaptive immunity of IL-17. Its role in regulating adipogenesis and glucose metabolism, along with strengthening TJ proteins like claudins and occludins, has previously been established (89). This further aligns with a plausible protective mechanism of LEN in promoting post-prandial glucose tolerance and epithelial integrity. More importantly, its protective role against metabolic syndrome has been demonstrated by limiting gut dysbiosis and LPS translocation (90). Further studies unraveling the role of specific T-cells in regulating IL-17 after RS treatment could provide more insights, as elevated levels of IL-17 could also induce inflammation. Nevertheless, we observed improvement in the intestinal integrity and inflammatory markers, further suggesting the protective role of these pulses-derived RS in the maintenance of normal glucose homeostasis under HFD-induced metabolic dysfunction (12).

In conclusion, our study provides novel insights pertaining to the prebiotic potential of dietary pulses-derived RS in positively modulating the gut microbiome architecture, strengthening the intestinal epithelial barrier integrity, and attenuating inflammation in a preclinical model of human microbiome and aging. Our findings also demonstrate that the prebiotic effects of RS differ among host sexes, further emphasizing the role of sexual dimorphism at microbiome, immune and metabolic level, which is crucial for establishing universality of health claims imparted by functional foods. However, the studied effects are specific to RS from specific beans and pulses, which is intelligible considering the structural differences associated with different pulse varieties. Nonetheless, based on the evidence accumulated from this study, LEN and CKP seemed to deliver more favorable outcomes in modulating gut microbiome and improving metabolic health. Still, further studies unraveling the changes in global microbial metabolomic, and transcriptomic arrays would be of great interest and importance in ascertaining the direct functional consequences of RS on host health. Future efforts will also be directed towards understanding the microstructural differences in RS to precisely target sexually dimorphic gut microbiome for preventing metabolic disorders.

## Data availability statement

The original contributions presented in the study are publicly available. This data can be found at: https://ncbi.nlm.nih.gov/bioproject/PRJNA902407.

## Ethics statement

This study was carried out in accordance with the recommendations and guidelines of the Institutional Animal Care and Use Committee. The protocol was approved by the Institutional Animal Care and Use Committee at Florida State University (PROTO202100008).

## Author contributions

SK performed experiments, analyzed data, and wrote manuscript. GP analyzed data and assisted in manuscript drafting. PS assisted in starch extractions, revised, and edited manuscript. BA provided critical inputs and advices on experimental design, data interpretation, reviewed, and edited manuscript. RN conceived the idea, supervised the study, analyzed and interpretated data, revised, and edited the manuscript. All authors reviewed and approved the final version of the manuscript.

## Funding

This work was supported by funding from the Pulse Crop Health Initiative program of the United States Department of Agriculture (USDA-ARS) to RN.

## Conflict of interest

The authors declare that the research was conducted in the absence of any commercial or financial relationships that could be construed as a potential conflict of interest.

## Publisher’s note

All claims expressed in this article are solely those of the authors and do not necessarily represent those of their affiliated organizations, or those of the publisher, the editors and the reviewers. Any product that may be evaluated in this article, or claim that may be made by its manufacturer, is not guaranteed or endorsed by the publisher.

## Footnotes

^1^ https://nuts.com/cookingbaking/beans/

^2^ https://earthmicrobiome.org/
