# Supplementary material for: Prebiotic mechanisms of resistant starches from dietary beans and pulses on gut microbiome and metabolic health in a humanized murine model of aging
Source: Front Nutr. 2023 Feb 7;10:1106463. doi: 10.3389/fnut.2023.1106463 (PMC9941547; doi:10.3389/fnut.2023.1106463)

Supplementary Table S1: Characteristics of pulses derived starches used in the study

Starch source	Yield (%)	Moisture (% DWB)	Granule size, average (Length x width)	Shape	Amylose (% w/w)	Resistant starch* (% DWB)		Reference[#]
						Raw	Cooked	
Pinto Bean	18.45±2.12	8.59±0.43	17.84 μm x 14.14 μm	Round-to-Oval	31.605	59.66	12.73	(1,2)
Black Eyed Pea	12.29±0.36	6.76±0.22	15.29μm x 10.93 μm	Round-to-Oval, irregular	33.063	72.36	13.08	(1,2)
Lentil	22.61±2.94	6.39±0.66	15.66 μm x 11.88 μm	Round-to-Oval, irregular	35.866	22.56	12.15	(1,2)
Chickpea	18.21±1.25	7.56±1.32	12.48 μm x 9.66 μm	Round-to-Oval, irregular	27.827	56.04	12.02	(1,2)

DWB: Dry weight basis

*Resistant starch determined using Megazyme kit # K-DSTRS

[#] (1) Sangokunle OO, Sathe SK, Singh P. Purified starches from 18 pulses have markedly different morphology, oil absorption and water absorption capacities, swelling power, and turbidity. *Starch-Stärke* (2020) 72:2000022.

(2) Sangokunle OO. Exploration of Purified Pulse Starches for Food and Health. [Dissertation]. Tallahassee: Florida State University (2021). https://purl.lib.fsu.edu/diginole/2021_Fall_Sangokunle_fsu_0071E_16809

Supplemental Table S2: Diet composition used in present study.

	CTL	PTB	BEP	LEN	CKP	INU
Ingredient, gm						
Casein	38.5	38.5	38.5	38.5	38.5	38.5
Fish Protein Isolate	8.5	8.5	8.5	8.5	8.5	8.5
Egg white	55	55	55	55	55	55
Beef, cooked, powdered	77	77	77	77	77	77
L-cystein	3	3	3	3	3	3
Corn starch	30	25	25	25	25	28
Wheat starch	195	168	168	168	168	180
Potato Starch	30	25	25	25	25	28
RS-PTB	0	44	0	0	0	0
RS-BEP	0	0	44	0	0	0
RS-LEN	0	0	0	44	0	0
RS-CKP	0	0	0	0	44	0
Inulin	5	5	5	5	5	45
Sucrose	205	205	205	205	205	205
Fructose	22	22	22	22	22	22
Cellulose	18	18	18	18	18	18
Menhaden oil	1	1	1	1	1	1
Butter, anhydrous	54	54	54	54	54	54
Lard	34	34	34	34	34	34
Flaxseed oil	1	1	1	1	1	1
Olive oil	28	28	28	28	28	28
t-BHQ	0.005	0.005	0.005	0.005	0.005	0.005
Mineral mix	10	10	10	10	10	10
Dicalcium phosphate	13	13	13	13	13	13
Calcium carbonate	5	5	5	5	5	5
Potassium Citrate	16	16	16	16	16	16
Vitamin mix	10	10	10	10	10	10
Biotin	0.1	0.1	0.1	0.1	0.1	0.1
Choline	2	2	2	2	2	2
Cholesterol	1.5	1.5	1.5	1.5	1.5	1.5
Kcal						
Protein	547	547	547	547	547	547
Carbohydrate	1824	1824	1824	1824	1824	1824
Fat	1289	1289	1289	1289	1289	1289
Total	3661	3661	3661	3661	3661	3661
Kcal%						
Protein	15	15	15	15	15	15
Carbohydrate	50	50	50	50	50	50
Fat	35	35	35	35	35	35
% Added RS	0	5	5	5	5	0
% Inulin	0.5	0.5	0.5	0.5	0.5	5
Kcal/g	4.2	4.2	4.2	4.2	4.2	4.2

Supplementary Table S3: List of primers used in current study for qPCR analysis

Gene name	Forward primer	Reverse primer	Reference#
18S	AGAAACGGCTACCACATCCA	CCCTCCAATGGATCCTCGTT	(3)
CLDN1	GGCTTCTCTGGGATGGATCG	CTTTGCGAAACGCAGGACAT	(3)
CLDN4	CGTAGCAACGACAAGCCCTA	TGTCCCCAGCAAGCAGTTAG	(3)
ZO1	AAGAAAAAGAATGCACAGAGTTGTT	GAAATCGTGCTGATGTGCCA	(3)
ZO2	AGCTTGTAGTTCTGAGCCGC	CCGACACGGCAATTCCAAAT	(3)
OCCL	CTGACTATGCGGAAAGAGTTGAC	CTGACTATGCGGAAAGAGTTGAC	(3)
JAM3	GCTGTGAGGTCGTTGCTCTA	AGTGGCACATCATTGCGGTA	(3)
IL1β	GAAATGCCACCTTTTGACAGTG	TGGATGCTCTCATCAGGACAG	(4)
IL10	TGGGTTGCCAAGCCTTATCG	TTCAGCTTCTCACCCAGGGA	(3)
IL17A	TCCCTCTGTGATCTGGGAAG	CTCGACCCTGAAAGTGAAGG	(5)
TNF-α	GATCGGTCCCCAAAGGGATG	TTTGCTACGACGTGGGCTAC	(3)

#(3) Miranda-Ribera A, Ennamorati M, Serena G, Cetinbas M, Lan J, Sadreyev RI, Jain N, Fasano A, Fiorentino M. Exploiting the zonulin mouse model to establish the role of primary impaired gut barrier function on microbiota composition and immune profiles. *Front Immunol*. (2019) 10:2233.

(4) Minato KI, Ohara A, Mizuno M. A proinflammatory effect of the β-glucan from *Pleurotus cornucopiae* mushroom on macrophage action. *Mediators Inflamm*. (2017) 22:2017.

(5) Wang Y, Xing F, Ye S, Xiao J, Di J, Zeng S, Liu J. Jagged-1 signaling suppresses the IL-6 and TGF-β treatment-induced Th17 cell differentiation via the reduction of RORγt/IL-17A/IL-17F/IL-23a/IL-12rb1. *Sci Rep*. (2015) 5:1-10.

Supplementary Figure 1S. Group-wise sex-specific differences in the **(A)** total gastrointestinal length, **(B)** colon length and **(C)** Cecum weight. (D) Liver weight for combined, male and female genders

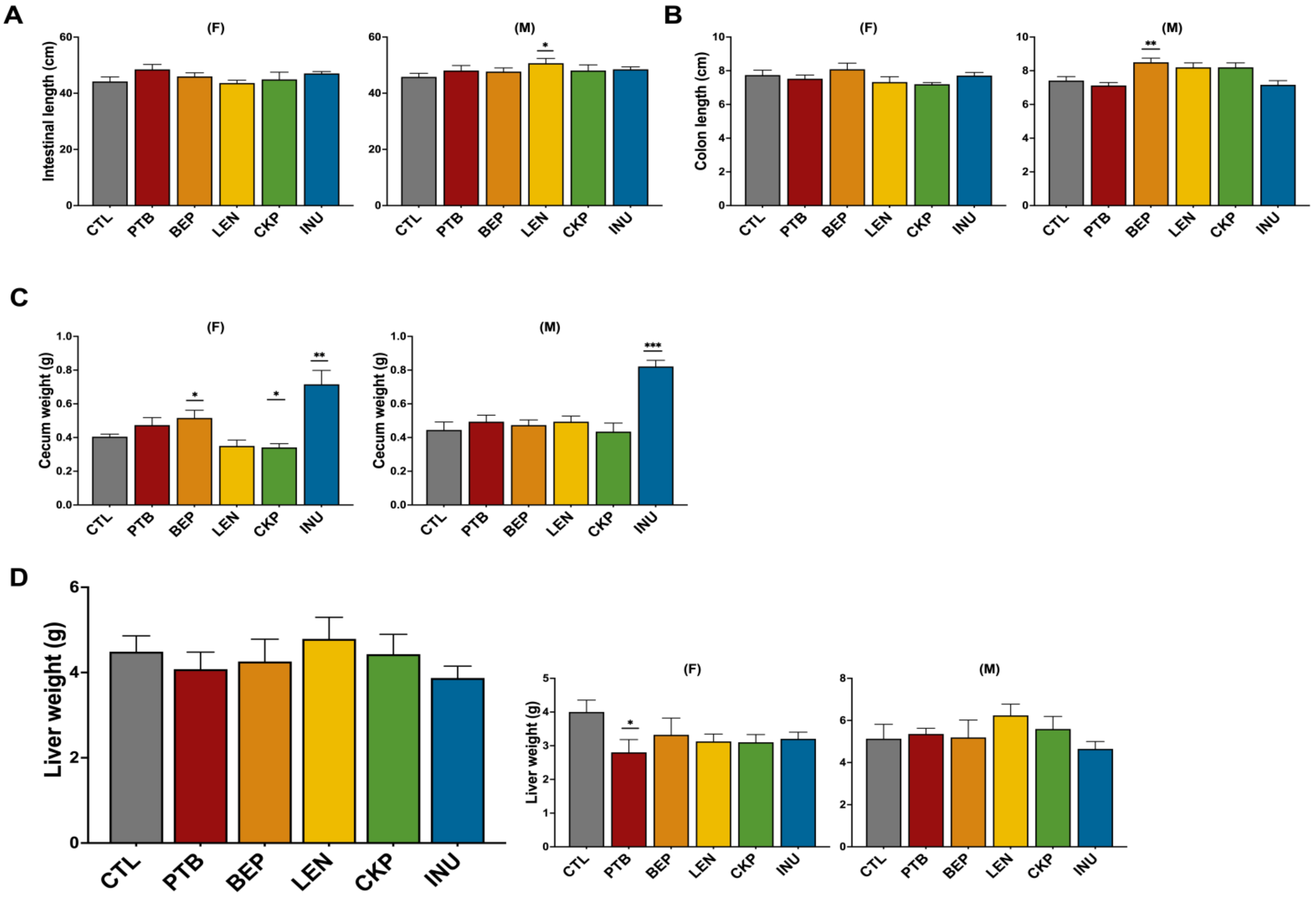

Supplementary Figure 2S. Hierarchical clustering dendrogram of the top 5 phyla, 10 families, and 20 genera based on Bray-Curtis distance metrics with the average method.

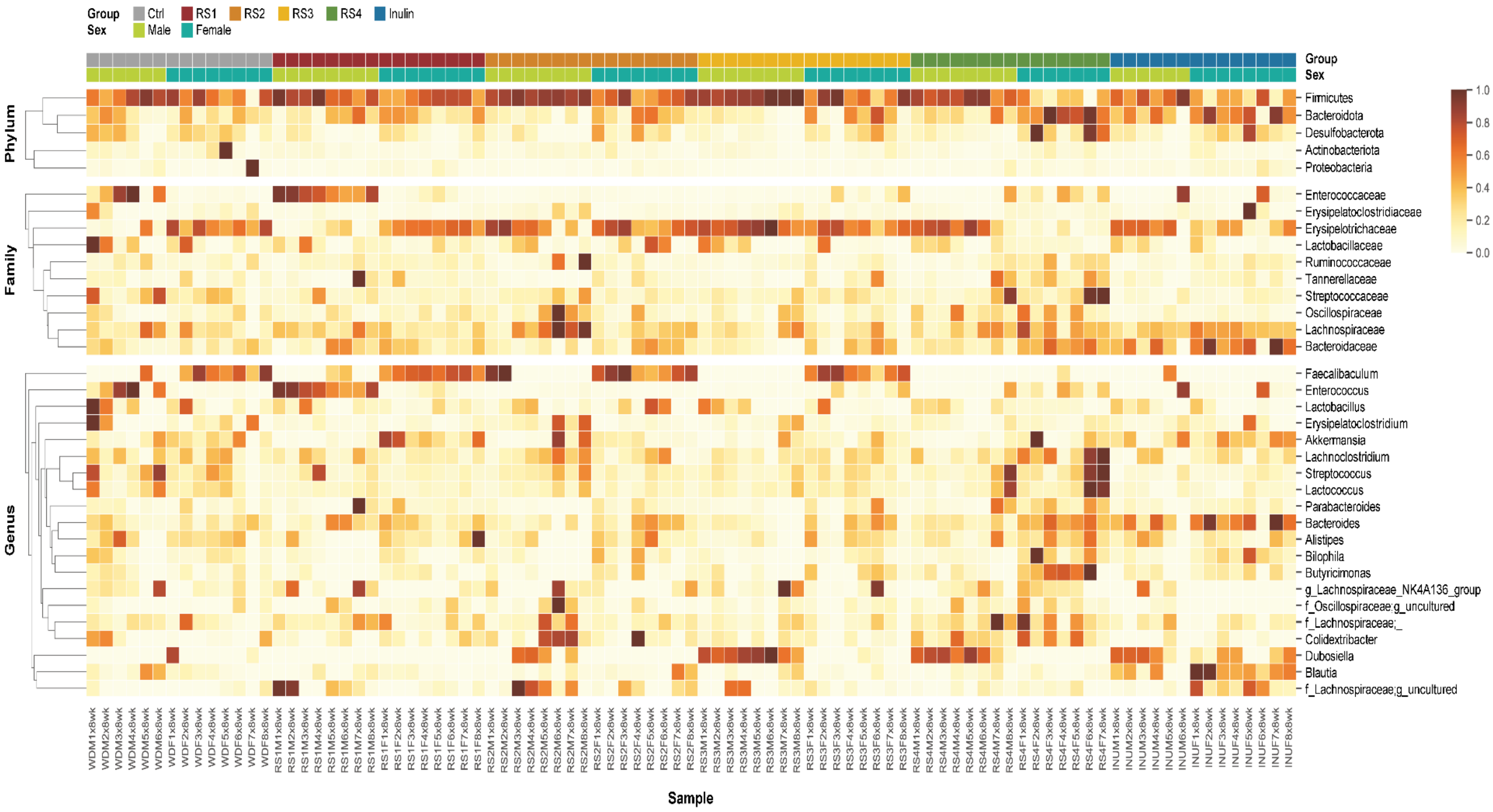

Supplementary Figure 3S. Random Forest prediction of six groups. **(A)** The confusion matrix of Random Forest classifier **(B)** Receiver Operating Characteristic (ROC) curve represents the classification accuracy and assessed by the area under curve (AUC). **(C)** Random Forest class probability histograms for six groups.

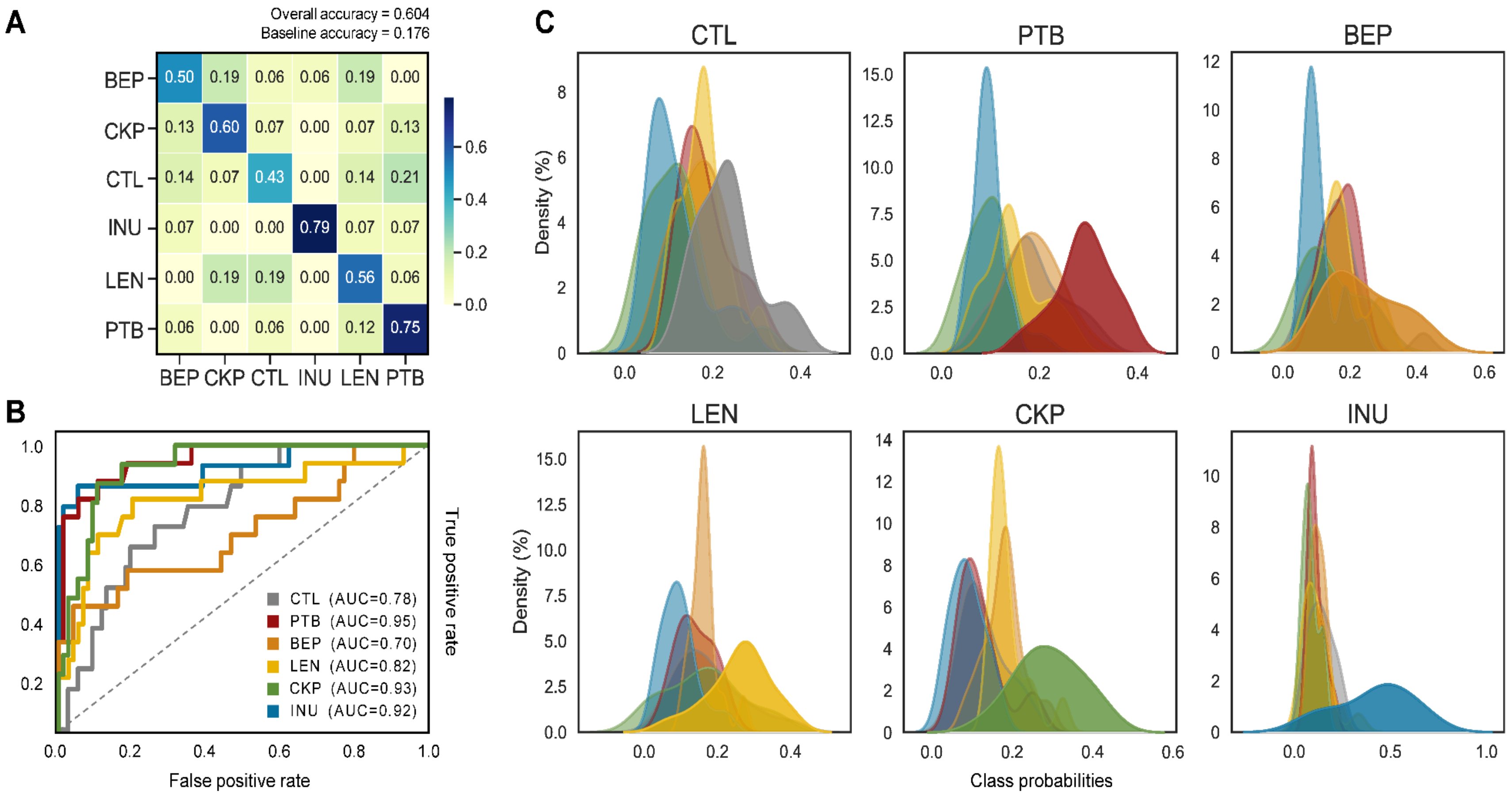

Supplementary Figure 4S. Differences in predictive KEGG functional capabilities based on 16S rRNA data was analyzed using PICRUSt and STAMP was used for pathway identification among different groups at **(A)** Level 2 and **(B)** Level 3.

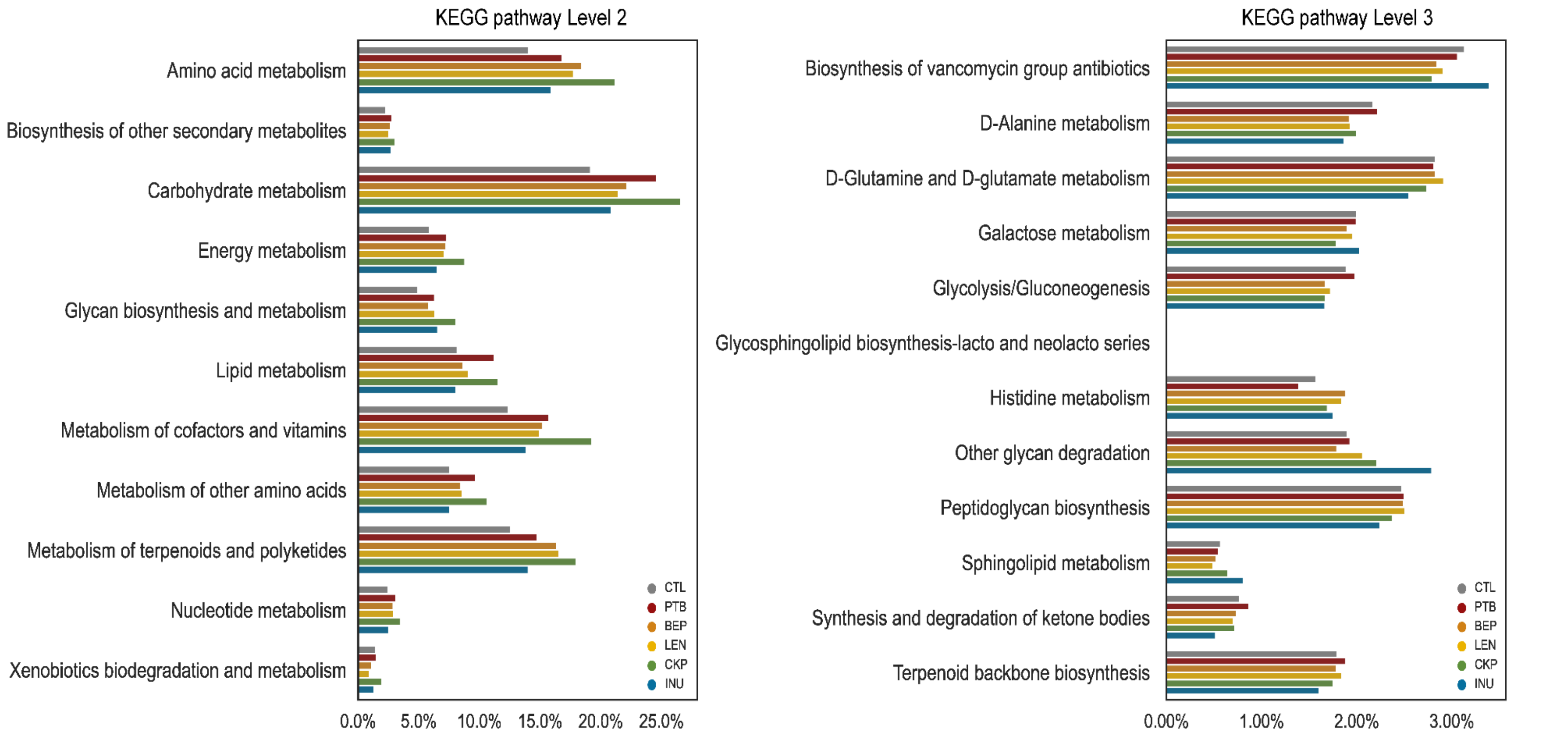

Supplement: Supplementary file 1 [file Data_Sheet_1.PDF]
